# Thermophile Lytic Enzyme Fusion Proteins that Target *Clostridium perfringens*

**DOI:** 10.3390/antibiotics8040214

**Published:** 2019-11-08

**Authors:** Steven M. Swift, Kevin P. Reid, David M. Donovan, Timothy G. Ramsay

**Affiliations:** 1 Animal Biosciences and Biotechnology Laboratory, United States Department of Agricultural (USDA), Agricultural Research Service, Baltimore Avenue, Beltsville, MD 10300, USA or sswift2010@gmail.com (S.M.S.); kreid414@gmail.com (K.P.R.); ddonovan0@yahoo.com (D.M.D.); 2 Contrafect Corporation., Yonkers, NY 10701, USA; 3 Department of Biology, Morgan State University, Baltimore, MD 21251, USA

**Keywords:** endolysin, peptidoglycan hydrolase, *Clostridium perfringens*, glucosaminidase, L-alanine-amidase

## Abstract

*Clostridium perfringens* is a bacterial pathogen that causes necrotic enteritis in poultry and livestock, and is a source of food poisoning and gas gangrene in humans. As the agriculture industry eliminates the use of antibiotics in animal feed, alternatives to antibiotics will be needed. Bacteriophage endolysins are enzymes used by the virus to burst their bacterial host, releasing bacteriophage particles. This type of enzyme represents a potential replacement for antibiotics controlling *C. perfringens*. As animal feed is often heat-treated during production of feed pellets, thermostable enzymes would be preferred for use in feed. To create thermostable endolysins that target *C. perfringens*, thermophile endolysin catalytic domains were fused to cell wall binding domains from different *C. perfringens* prophage endolysins. Three thermostable catalytic domains were used, two from prophage endolysins from two *Geobacillus* strains, and a third endolysin from the deep-sea thermophilic bacteriophage *Geobacillus* virus E2 (GVE2). These domains harbor predicted L-alanine-amidase, glucosaminidase, and L-alanine-amidase activities, respectively and degrade the peptidoglycan of the bacterial cell wall. The cell wall binding domains were from *C. perfringens* prophage endolysins (Phage LYtic enzymes; Ply): PlyCP18, PlyCP10, PlyCP33, PlyCP41, and PlyCP26F. The resulting fifteen chimeric proteins were more thermostable than the native *C. perfringens* endolysins, and killed swine and poultry disease-associated strains of *C. perfringens*.

## 1. Introduction

*Clostridium perfringens* is a Gram-positive, spore forming, anaerobic bacterium, commonly present in the intestines of humans and animals. Spores of the pathogen can persist in soil [1], feces [2] or the environment [3]. The bacterium causes many severe infections of animals and humans, including food poisoning, gas gangrene, necrotic enteritis and non-foodborne gastrointestinal infections in humans [4]. *C. perfringens* has been estimated to be the third leading cause of foodborne illness in the U.S. [5]. *C. perfringens* strains have commonly been sorted into one of five types, A, B, C, D, and E, based on the types of toxins that the cells produce. Each type has been associated with gastro-intestinal disease in different animals [4].

In swine, *C. perfringens* most commonly causes disease in neonatal piglets [6]. *C. perfringens* type C can cause necrotic enteritis in piglets within the first five days of life, often resulting in high mortality rates in infected litters [7,8]. Additionally, piglets of a few days of age can suffer from diarrhea commonly caused by *C. perfringens* type A [9]. While this may not be fatal, the loss of weight gain can result in economic losses.

In chickens, *C. perfringens* type A is believed to be a causative agent of necrotic enteritis, the most common and financially devastating bacterial disease in modern broiler flocks [10]. Although the clinical illness is usually very short, mortality in an unprotected poultry flock can be devastating. Indeed, often the only sign of necrotic enteritis in a flock is a sudden increase in mortality. In addition to increased mortality, necrotic enteritis may present as birds with depression, ruffled feathers, and dark diarrhea. The disease can persist in a flock for several days, with mortality of 1% per day [11].

The use of antibiotics as animal growth promoters (AGP) has been banned in the European Union (EU), January 2006 (Regulation 1831/2003/EC), due to concerns about the practice leading to a rise in antibiotic resistance in bacteria. In 2017, the United States Food and Drug Administration (FDA) released guidelines limiting the use of antibiotics in animal feed for AGP purposes [12,13]. Without traditional antibiotics for the prevention of necrotic enteritis and other diseases caused by *C. perfringens*, such diseases could potentially become a far greater problem for the swine and poultry industry. Therefore, alternatives to traditional antibiotics are needed, which are effective in preventing and treating disease caused by *C. perfringens*, especially *C. perfringens* that affect swine and poultry. Additionally, should traditional antibiotics fail in a medical setting, these alternatives may be useful for treating infected humans.

Bacteriophage are viruses that infect bacteria, and produce lytic enzymes called endolysins (or lysins) during the infective process. The holin, another phage protein, facilitates endolysins crossing the inner membrane to reach the peptidoglycan of the cell wall. Endolysins are peptidoglycan hydrolases, and break open the host cells by degrading the peptidoglycan, resulting in osmolysis and the release of the replicated viral particles. Gram positive bacteria lack an outer membrane, and the peptidoglycan of the cell wall is exposed to the action of endolysins applied externally (reviewed in [14]). For these bacteria, endolysins can lyse them when exposed from the outside. Since the site of action for an endolysin is external to the pathogen, this avoids some of the intracellular drug resistance mechanisms, e.g., efflux pumps. Additionally, the phage and host have co-evolved, allowing the phage endolysin to recognize target sites in the cell wall that are difficult for the bacterium to mutate. Thus, it is believed that phage endolysins are highly refractory to resistance development [15]. This characteristic makes endolysins a good source of anti-bacterial agents against Gram-positive bacteria, like *C. perfringens*. 

In the field of human therapeutics, two lysins, SAL200 and CF-301, are in clinical trials in the U.S. for the treatment of patients with *Staphylococcus aureus* bacteremia, or bloodstream infections. These trials are listed at ClinicalTrials.gov, with the identifier codes NCT03089697 for SAL200 and NCT03163446 for CF-301. These are drug add-on trials, where the patients receive standard of care treatment antibiotics with or without the lysin drug. Another lysin, named Staphefekt, has been tested in the Netherlands as a topical treatment for *Staphylococcus aureus*-associated atopic dermentitis [16]. No endolysin in animal feed studies were found from a search of PubMed at NCBI. However, another type of peptidoglycan hydrolase, hen egg white lysozyme, was tested in liquid feed (not feed pellets) for piglets, and shown to improve animal growth and to lower counts of pathogenic bacteria [17,18].

The production of animal feed pellets usually involves heat-treatment of the feed [19]. This treatment entails conditioning the mixture of feed ingredients with steam at temperatures up to 95 °C, with exposure times of 20 seconds to 4 min in a feed pellet mill [20]. Thus, for endolysins to be incorporated into animal feed pellet production, they should be thermostable or thermotolerant. It is known that Gram positive phage endolysins are modular proteins (usually), with N-terminal catalytic (CAT) and C-terminal cell wall binding (CWB) domains. CAT domains can often be redirected to target a different species by swapping CWB domains [21]. Previously, we have shown that the CAT domain from a thermostable endolysin of a different species could be re-targeted against *C. perfringens* [22]. Thermophilic bacteria produce thermostable enzymes whose CAT domains can potentially be re-targeted against *C. perfringens*. Thermophilic bacteria genomes were examined for prophages (bacteriophage genomes inserted into the bacterial genome). These prophage regions typically encode an endolysin gene. Two prophage endolysins from thermophilic *Geobacillus* bacteria, identified by a bioinformatics search, along with the previously identified phage lytic enzyme (Ply) PlyGVE2 endolysin, were used here. In this study, we report on a set of chimeric lysins created by fusing thermophile endolysin CAT domains with *C. perfringens*-targeting CWB domains. 

## 2. Results

### 2.1. Peptidoglycan Structure and Endolysin Cleavage Sites

**Figure 1:**
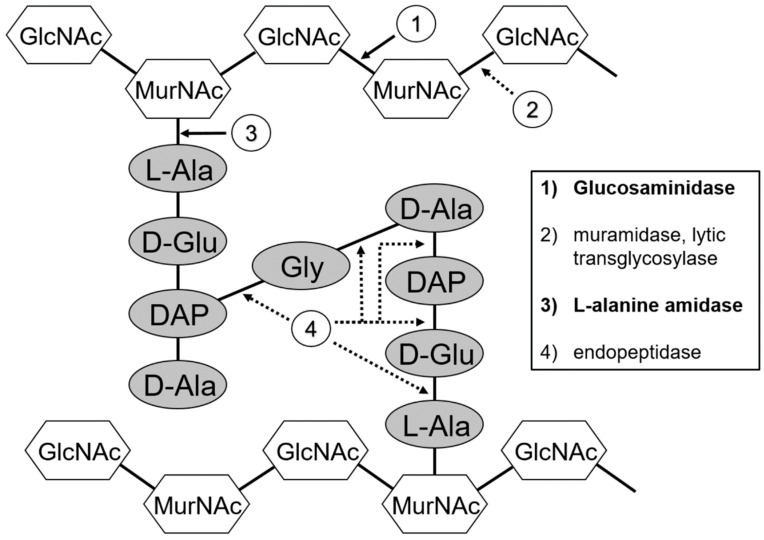
Peptidoglycan structure and sites of hydrolase cleavage. (1) Glucosaminidases cleave between N-acetyl glucosamine (GlcNac) and N-acetyl muramic acid (MurNac). (2) Muramidases (lysozymes) and lytic transglycosylases cleave between MurNac and GlcNac. (3) L-alanine-amidases cleave between the MurNac and the first amino acid of the peptide stem, an alanine. (4) Endopeptidases cleave between the peptide bonds in the peptide stem, or in the cross-bridge that links the stems.

Bacterial peptidoglycan has a complex structure, with a sugar backbone of alternating units of N-acetyl glucosamine (GlcNac) and N-acetyl muramic acid (MurNac) residues, cross-linked by oligopeptide attachments (the peptide stem) at the MurNac, with peptide stems linked by a peptide cross-bridge. The *C. perfringens* peptidoglycan schematic is shown in Figure 1 [23]. Endolysins have evolved a modular design to deal with this complexity, with a C-terminal CWB domain and an N-terminal CAT domain. One protein can harbor one or two CAT domains, each with a different peptidoglycan digestion activity [14,24]. Endolysin CAT domains can be sorted into three broad categories, endopeptidase, glycosidase and amidase, that target different bonds in the peptidoglycan structure. Each CAT domain is small (70–200 amino acids). Any one of these domains is sufficient to lyse the bacterial target cell. Gram-positive cell walls have at least 10 to 20 layers of peptidoglycan, compared to one to three layers for Gram-negative cells [25].

### 2.2. Discovery and Bioinformatic Analysis of Thermophile Endolysins

Gram-positive thermophilic bacteria genomes were examined at the Integrated Microbial Genomes (IMG) website (http://img.jgi.doe.gov/) for potential prophage endolysin sequences. Genes encoding potential endolysin proteins were identified adjacent to predicted bacteriophage genes. Two predicted endolysin genes were found in the genomes of *Geobacillus* strains Y412MC61 and Y4.1MC1. According to the IMG database, both strains were isolated from hot springs at the Yellowstone National Park, USA, and grown at 70 °C in the laboratory. As these thermophilic bacteria grow at elevated temperatures, it is likely that their proteins, including endolysins, are thermostable. Each endolysin was predicted by the NCBI Conserved Domain search engine [26] to have different enzymatic activities, and cleave a different bond in the peptidoglycan (Figure 1); one is an L-alanine amidase that targets the bond between the peptide stem and the glycan, and the other is a glucosaminidase that targets the glycan polymer. Both endolysins target peptidoglycan bonds that are highly conserved across bacteria species.

**Figure 2:**
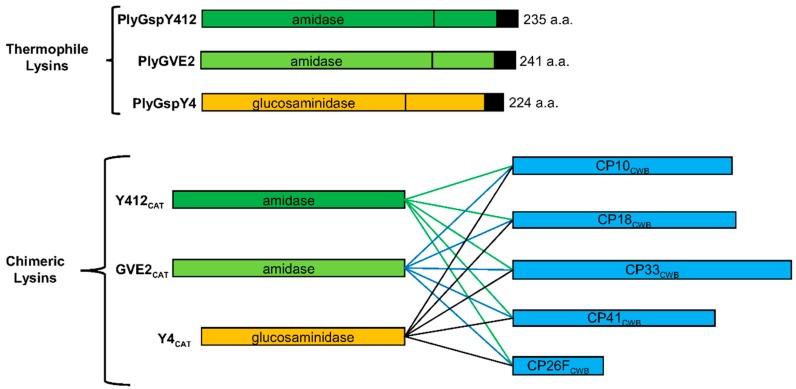
Thermophile-derived chimeric recombinant lysins schematic. The thermophile endolysins, PlyGspY412, PlyGspY4, and PlyGVE2, supply the N-terminal catalytic (CAT) domains, GVE2_CAT_, Y412_CAT_ and Y4_CAT_, respectively. The C-terminal CWB domain comes from one of several *C. perfringens* endolysins (PlyCP10, PlyCP18, PlyCP33, PlyCP41, PlyCP26F). The recombinant proteins have the amino acids LEHHHHHH added to the C-terminus to facilitate purification by nickel-affinity chromatography; those with CP18_CWB_ have VEHHHHHH. GVE2_CAT_-CP26F_CWB_ is the previously published lysin PlyGVE2CpCWB [22].

The first endolysin, named here as PlyGspY412 (IMG gene ID: 646372128; Genbank accession ACX77322), was found in the genome of *Geobacillus* sp. Y412MC61. It has an N-terminal L-alanine amidase domain, belonging to a subgroup or family defined by PFAM (http://pfam.xfam.org/) as amidase_3 (Figure 2), which cleaves the peptidoglycan bond between the MurNAc glycan residue and the alanine of the peptide stem (Figure 1). In the C-terminal half, there is a sporulation-related repeat (SPOR) domain, PFAM code PF05036, which is a peptidoglycan binding domain consisting of two 35-residue repeats, that is associated with proteins involved in sporulation, and is also found in some endolysin proteins. A BlastP search at NCBI website of the PlyGspY412 protein sequence against the *Clostridium perfringens* (taxid:1502) dataset yielded three hits at 34% identity (WP_115634784, WP_025647622, WP_070956814), all of which were L-alanine amidases with C-terminal SPOR domains. 

The other new endolysin, named here as PlyGspY4 (IMG gene ID: 649721886; Genbank accession ADP73474), was found in the genome of *Geobacillus* sp. Y4.1MC1. This bacteria was collected from a 88 °C water outflow from a hot spring [27]. PlyGspY4 has a predicted N-terminal glucosaminidase CAT domain (PFAM code PF01832) and a C-terminal LysM (PFAM code PF01476) peptidoglycan binding domain (Figure 2). BlastP analysis of PlyGspY4 protein sequence against the *C. perfringens* data set at NCBI yielded 88 hits, with the top three hits being glucosaminidase enzymes and having 39%–40% identity (WP_124044957, WP_110003240, WP_110021405).

A third endolysin comes from the *Geobacillus* virus E2, or GVE2, which was isolated from a deep-sea hydrothermal vent, and can infect *Geobacillus* sp. E263 [28]. The GVE2 phage endolysin, named here as PlyGVE2 (Genbank accession YP_001285830), was previously characterized and shown to be active at temperatures up to 80 °C [29]. PlyGVE2 provided the CAT domain for the fusion protein, PlyGVE2CpCWB, in a previous publication [22]. Similar to PlyGspY412, PlyGVE2 has an N-terminal amidase domain, and a C-terminal SPOR domain. Clustal Omega alignment of the protein sequences for PlyGVE2 and PlyGspY412 yielded a 60.7% identity between the two endolysins. BlastP analysis of PlyGVE2 against the *C. perfringens* data set at NCBI yielded 41 hits, with the top three hits having 37% identity, which were the same hits found for PlyGspY412.

Other predicted thermophile endolysins, initially included in our study, were removed due to absence of lytic activity against *C. perfringens*, or due to a failure to express soluble protein. The CAT domain from a predicted endopeptidase from *Geobacillus vulcani* PSS1 (Genbank accession WP_084177458) fused to CP18_CWB_ was successfully expressed and purified, but did not have lytic activity against *C. perfringens* (data not shown). Additionally, constructs containing a CAT domain from a predicted L-alanine amidase endolysin from *Clostridium thermocellum* ATCC 27405 (Genbank accession YP_001038028) were insoluble when expressed in *E. coli* (data not shown).

### 2.3. Chimeric Lysins Description, Expression, and Purification

The thermophile endolysin gene constructs were synthesized (Genscript) with *E. coli*-codon bias. These synthesized genes served as templates for PCR, to amplify the sequences of the CAT domains and clone them into the pET21a plasmid for expression in BL21(DE3) *E. coli*. The *E. coli*-codon optimized sequences for the CWB domains from several *C. perfringens* prophage endolysins—PlyCP10, PlyCP18, PlyCP33, PlyCP41, and PlyCP26F—were then PCR amplified and cloned into the plasmids with the thermostable CAT domains (GVE2_CAT_, Y412_CAT_, and Y4_CAT_) sequences, to make the 15 lysin fusions (Figure 2) using standard molecular biology techniques. The Genbank accessions numbers for the *C. perfringens* lysins are as follows: PlyCP26F (AEA86246), PlyCP10 (AQS60701), PlyCP18 (AQS60703), PlyCP33 (AQS60704), and PlyCP41 (AQS60702). All five *C. perfringens* lysins target *C. perfringens* strain Cp39 (US patent application 20180195055; [30,31]). All recombinant plasmids were sequenced to confirm identity and cloning integrity. The peptide sequences for the chimeric lysins are available in Supplementary Figure S1.

Five different CWB domains were used to increase the likelihood of creating an active fusion protein. PlyCP26F has a SPOR domain (PFAM code PF05036) in its CWB; domain predicted by HMMScan [32]. The rest of these lysins have SH3_3-type binding domains (PFAM code PF08239) in their CWBs, with differences in their sequences and in the spacing of the domains within the CWB regions of the proteins. These CWBs potentially have a different binding affinity to *C. perfringens*, as the endolysins they came from have differences in activity against different strains of *C. perfringens* (US patent application 20180195055; [30,31]). Therefore, combining the three thermophile derived CAT domains with the five CWBs regions should increase the odds that one or more fusion proteins would be active, and potentially thermostable.

**Figure 3:**
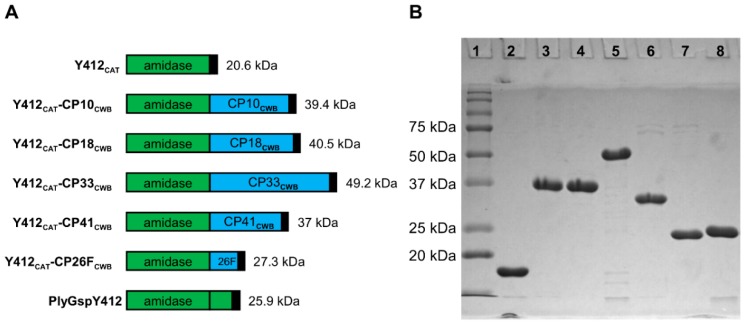
PlyGspY412 derived recombinant lysins schematics and SDS-PAGE. (**A**) Schematics: PlyGspY412 supplied the N-terminal CAT domain (green), Y412_CAT_. C-terminal CWB domains (blue) were derived from one of five *C. perfringens* endolysins (PlyCP10, PlyCP18, PlyCP33, PlyCP41, PlyCP26F). Black box indicates the C-terminal 6 x His domain. Each recombinant protein has a C-terminal LEHHHHHH to facilitate purification by nickel-affinity chromatography; those with CP18_CWB_ have VEHHHHHH (as described in Supplementary Data). (**B**) SDS-PAGE of Y412_CAT_ fusion proteins purified by nickel chromatography. Lane 1 is Precision Plus Protein standard (Bio-Rad). The other lanes are as follows: lane 2, Y412_CAT_; lane 3, Y412_CAT_-CP10_CWB_; lane 4, Y412_CAT_-CP18_CWB_; lane5, Y412_CAT_-CP33_CWB_; lane 6, Y412_CAT_-CP41_CWB_; lane 7, Y412_CAT_-CP26F_CWB_; lane 8, PlyGspY412.

**Figure 4:**
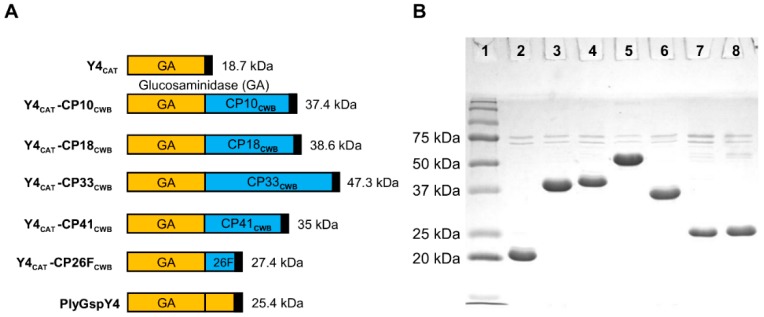
PlyGspY4-derived recombinant lysins purity via SDS-PAGE. (**A**) Schematics: PlyGspY4 supplied the N-terminal CAT domain (orange), Y4_CAT_. The C-terminal CWB domain (blue) is derived from one of five *C. perfringens* endolysins (PlyCP10, PlyCP18, PlyCP33, PlyCP41, PlyCP26F). Black box indicates the C-terminal 6 x His domain. (**B**) SDS-PAGE of Y4_CAT_ fusion proteins purified by nickel chromatography. Lane 1 is Precision Plus Protein standard (Bio-Rad). The other lanes are as follows: lane 2, Y4_CAT_; lane 3, Y4_CAT_-CP10_CWB_; lane 4, Y4_CAT_-CP18_CWB_; lane5, Y4_CAT_-CP33_CWB_; lane 6, Y4_CAT_-CP41_CWB_; lane 7, Y4_CAT_-CP26F_CWB_; lane 8, PlyGspY4.

**Figure 5:**
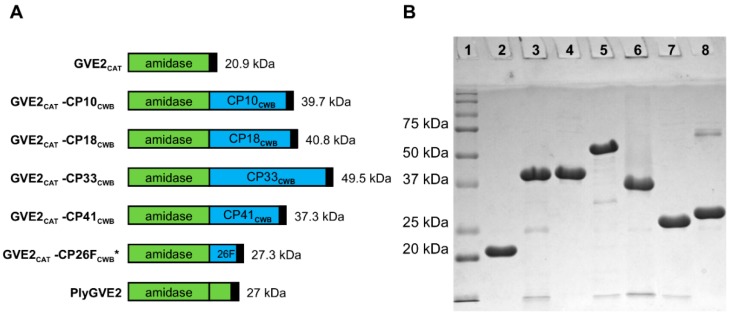
PlyGVE2-derived recombinant lysins schematics and SDS-PAGE. (**A**) Schematics, PlyGVE2 supplied the N-terminal CAT domain (green), GVE2_CAT_, and the C-terminal CWB domain (blue) comes from one of several *C. perfringens* endolysins (PlyCP10, PlyCP18, PlyCP33, PlyCP41, PlyCP26F). Black box indicates the C-terminal 6 x His domain. GVE2_CAT_-CP26F_CWB_ * is the previously published lysin PlyGVE2CpCWB. (**B**) SDS-PAGE of GVE2_CAT_ fusion proteins purified by nickel chromatography. Lane 1 is Precision Plus Protein standard (Bio-Rad). The other lanes are as follows: lane 2, GVE2_CAT_; lane 3, GVE2_CAT_-CP10_CWB_; lane 4, GVE2_CAT_-CP18_CWB_; lane5, GVE2_CAT_-CP33_CWB_; lane 6, GVE2_CAT_-CP41_CWB_; lane 7, GVE2_CAT_-CP26F_CWB_; lane 8, PlyGVE2.

Proteins were expressed in BL21 (DE3) *E. coli* and purified by their His Tag, using nickel affinity chromatography. Purity of >95% is apparent from each of the recombinant proteins in the SDS-PAGE, (Figure 3, Figure 4 and Figure 5). PlyGspY412 and most of the chimeric proteins containing its CAT domain, Y412_CAT_-CP33_CWB_, migrated in the gel (Figure 3) one to three kilodaltons (kDa) faster than their predicted MW: Y412_CAT_-CP10_CWB_; 39.4 kDa, Y412_CAT_-CP18_CWB_; 40.5 kDa, Y412_CAT_-CP41_CWB_; 37 kDa, Y412_CAT_-CP26F_CWB_; 27.3 kDa, and PlyGspY412; 25.9 kDa. Y412_CAT_-CP33_CWB_ migrated slightly slower, around 52 kDa, in the gel, versus the 49.2 kDa predicted from its sequence.

As seen in Figure 4, all Y4_CAT_-containing proteins, except Y4_CAT_-CP33_CWB_, migrated in the gel within one to three kDa of their predicted MW: Y4_CAT_; 18.7 kDa, Y4_CAT_-CP10_CWB_; 37.4 kDa, Y4_CAT_-CP18_CWB_; 38.6 kDa, Y4_CAT_-CP41_CWB_; 35 kDa, Y4_CAT_-CP26F_CWB_; 27.4 kDa, and PlyGspY4; 25.4 kDa. Y4_CAT_-CP33_CWB_ ran closer to 55 kDa on the gel, larger than the sequence prediction of 47.3 kDa.

In Figure 5, all purified GVE2_CAT_-containing proteins, except GVE2_CAT_-CP33_CWB_, migrated in the gel within one to three kDa of their predicted MW: GVE2_CAT_; 20.9 kDa, GVE2_CAT_-CP10_CWB_; 39.7 kDa, GVE2_CAT_-CP18_CWB_; 40.8 kDa, GVE2_CAT_-CP41_CWB_; 37.3 kDa, GVE2_CAT_-CP26F_CWB_; 27.3 kDa, and PlyGVE2; 27 kDa. GVE2_CAT_-CP33_CWB_ has a predicted size of 49.5 kDa, but ran closer to 55 kDa on the gel. 

### 2.4. Lysis of C. perfringens by the Chimeric Lysins

**Figure 6:**
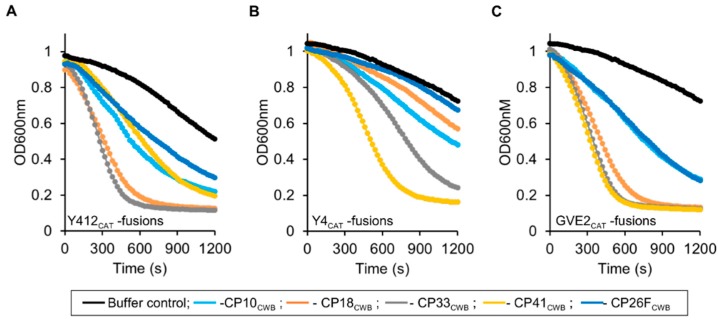
Turbidity reduction assay activity of Y412_CAT_, Y4_CAT_, GVE2_CAT_, and their fusions. (**A**) Y412_CAT_ and fusions. (**B**) Y4_CAT_ and fusions. (**C**) GVE2_CAT_ and fusions. Lines are as follows: buffer, black; fusion to CP10_cwb_, light blue; fusion to CP18_cwb_, orange; fusion to CP33_cwb_, gray; fusion to CP41_cwb_, yellow; fusion to CP26F_cwb_ dark blue. Enzymes were diluted into 50 mM NaH_2_PO_4_ pH 7.0 and mixed 1:1 with *Clostridium perfringens* cells in water. Turbidity was read as optical density (OD) at 600 nm shown over 20 min. The decrease in optical density (OD) represents lysis of cells in suspension. Final enzyme concentration was 0.005 mg/mL. Representative (single well) data shown.

Testing for lytic activity of the recombinant lysins was done by turbidity reduction assay. Lysins were mixed with mid-log phase *Clostridium perfringens* cells (strain Cp39), and the optical density, the turbidity, was reduced by the lysis of the cells by the recombinant proteins in phosphate buffer. The cells in buffer alone (buffer control), have decreasing turbidity overtime, likely because of lysis from the handling to put cells into buffer, exposure to buffer, and/or exposure to air, as these bacteria are anaerobic. The greater loss of turbidity that seen for the buffer control is indicative of lytic activity. Figure 6A shows the degree of lytic activity of the recombinant lysins derived from PlyGspY412, harboring the Y412_CAT_ (the amidase CAT domain). Y412_CAT_-CP33_CWB_ had the steepest curve, indicating the highest rate of turbidity reduction. Lytic activity was as follows (highest to lowest): Y412_CAT_-CP33_CWB_ > Y412_CAT_-CP18_CWB_ > Y412_CAT_-CP10_CWB_ = Y412_CAT_-CP41_CWB_ > Y412_CAT_-CP26F_CWB_ Figure 6A. PlyGspY412 and Y412_CAT_ were inactive against Cp39 cells (see Supplementary Figure S2, Table S1).

Testing of the PlyGspY4 derived CAT domain, Y4_CAT_, and its fusion proteins was done by standard turbidity reduction assay. As seen in Figure 6B, lytic activity was as follows (highest to lowest): Y4_CAT_-CP41_CWB_ > Y4_CAT_-CP33_CWB_ > Y4_CAT_-CP10_CWB_ > Y4_CAT_-CP18_CWB_. Y4_CAT_-CP26F_CWB_ was inactive against Cp39 cells, as seen by its line adjacent to the buffer control line at the top of the plot in Figure 6B. Interestingly, Y4_CAT_-CP41_CWB_ appears to be more active compared to the other Y4_CAT_-fusions, whereas the Y412_CAT_-CP41_CWB_ fusion falls into the middle of the Y412_CAT_-fusions in terms of activity in this assay. PlyGspY4 and Y4_CAT_ were inactive against Cp39 cells (see Supplementary Figure S2, Table S1).

The degree of lytic activity of the recombinant lysins derived from PlyGVE2, harboring the GVE2_CAT_ (the amidase CAT domain), are shown in Figure 6C. GVE2_CAT_-CP41_CWB_ (Gve2-41) and GVE2_CAT_-CP33_CWB_ (Gve2-33) had the steepest curves, indicating the highest rate of turbidity reduction. Lytic activity was as follows (highest to lowest): GVE2_CAT_-CP41_CWB_ = GVE2_CAT_-CP33_CWB_ > GVE2_CAT_-CP18_CWB_ > GVE2_CAT_-CP10_CWB_ = GVE2_CAT_-CP26F_CWB_. GVE2_CAT_ and PlyGVE2 had trace activity against Cp39 cells (see Supplementary Figure S2, Table S1).

**Figure 7:**
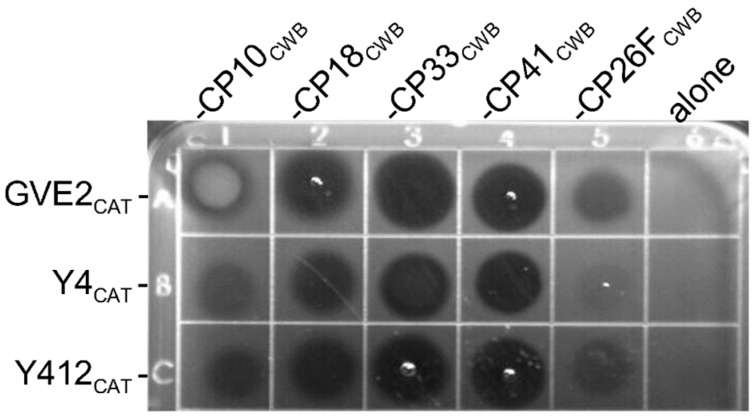
Plate lysis assay of the recombinant chimeric lysins. *C. perfringens* Cp39 cells were embedded in semisolid BYC agar, and then 10 micrograms of each lysin was spotted on top of the solidified agar and allowed to dry. The plate was incubated at 37 °C for 2 h. Clear zones indicate lytic activity of the lysins. The CAT domains alone, not fused to a binding domain, did not display activity (final column). Row A represents the chimeric lysins based on the PlyGVE2 (GVE2_CAT_) CAT domain fused to the CWB from PlyCP10, PlyCP18, PlyCP33, PlyCP41, and PlyCP26F. Row B represents the chimeric lysins based on the CAT domain from PlyGspY4 (Y4_CAT_) fused to the same binding domains. Row C represents the chimeric lysins based on the CAT domain from PlyGspY412 (Y412_CAT_) fused to the same binding domains.

Due to the fact that all enzymes do not yield quantitatively equivalent activity in all peptidoglycan hydrolase assays [33], it is useful to show similar relative activity between enzymes in more than one assay. Another test of lytic activity is the plate lysis assay. All chimeric lysins containing a *C. perfringens*-targeting CWB domain had activity and created a clear zone on the plate (Figure 7, rows A–C for columns 1–5). Fusions to the binding domains from PlyCP18, PlyCP33 and PlyCP41 appeared most active, (Figure 7, columns 2, 3, and 4, respectively), whereas fusions to the binding domain from PlyCP26F had low to miniscule activity in this assay, and made faint to very faint clearings (Figure 7, column 5). However, the CAT domains without a binding domain were not active and did not create clear zones (Figure 7, column 6). The GVE2_CAT_-CP10_CWB_ fusion (Figure 7, row A column 1) displayed a white discoloration on the plate, which may represent protein precipitation. It is apparent from these experiments, plate lysis and turbidity reduction assays (TRA), that not all CWB domains are equivalent in their ability to redirect the thermophile lysin CAT domain. The chimeric lysins, with CWB domains from PlyCP41, PlyCP33, and PlyCP18, were more active against *C. perfringens* than those using the binding domains from PlyCP10 or PlyCP26F. Interestingly, Y412_CAT_-CP41_CWB_ appears more active in this assay, relative to the other Y412_CAT_-containing lysins (Figure 7, row C), than it did in the TRA assay (Figure 6A).

### 2.5. Thermostability of the Chimeric Lysins

**Figure 8:**
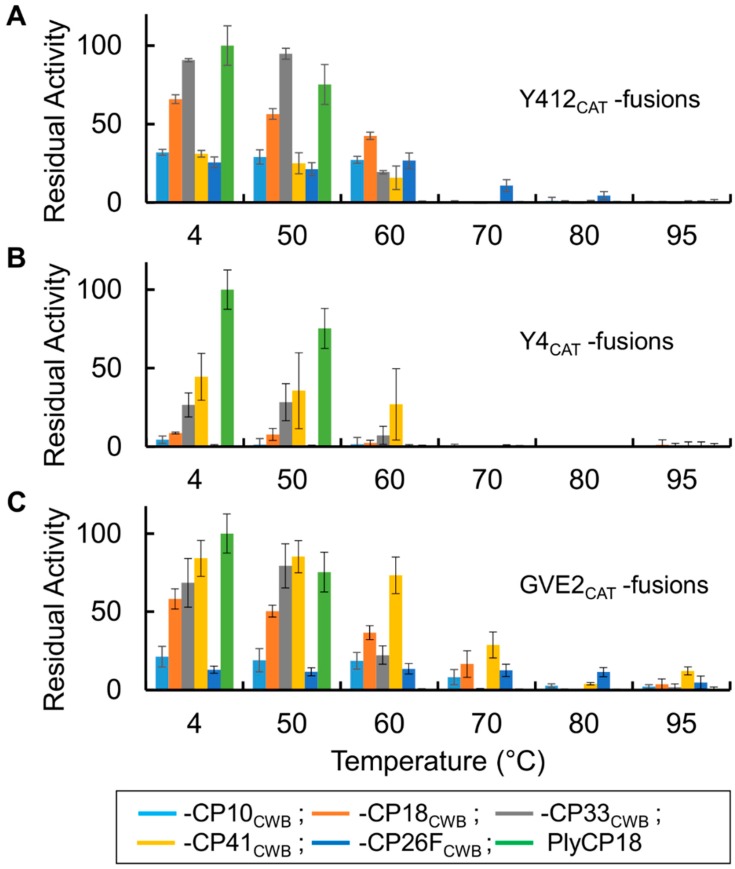
Thermostability of lysin fusion proteins. (**A**) PlyGspY412 (Y412_CAT_) derived fusion proteins compared to PlyCP18. (**B**) PlyGspY4 (Y4_CAT_) derived fusion proteins. (**C**) PlyGVE2 (GVE2_CAT_) derived fusion proteins. Bars are as follows: fusion to CP10_CWB_, light blue; fusion to CP18_CWB_, orange; fusion to CP33_CWB_, gray; fusion to CP41_CWB_, yellow; fusion to CP26F_CWB_ (PlyGVE2CpCWB), dark blue; and PlyCP18, green. Proteins were incubated 15 min at the indicated temperature (X-axis), cooled on ice, and then assayed at 40 °C for residual activity. On the Y-axis is the residual activity relative to the activity of PlyCP18 (4 °C data point).

Most of the thermophile fusion proteins are more tolerant of heating than PlyCP18 (Figure 8). PlyCP18 lost activity after incubation at 60 °C. All the Y412_CAT_ fusions retained substantial activity after the 60 °C heat challenge (Figure 8A), and Y412_CAT_-CP26F_CWB_ retained activity after heat treatment at 70 °C. Of the Y4_CAT_ derived fusion proteins, only Y4_CAT_-CP41_CWB_ retain substantial activity after incubation at 60 °C (Figure 8B). The GVE2_CAT_ derived fusion proteins showed substantial residual activity after being exposed to elevated temperatures (Figure 8C). All the GVE2_CAT_ fusions showed activity after incubation at 60 °C while GVE2_CAT_-CP10_CWB_, GVE2_CAT_-CP18_CWB_, GVE2_CAT_-CP41_CWB_, and the published fusion PlyGVE2CpCWB (GVE2_CAT_-CP26F_CWB_) showed activity after 70 °C heat treatment. The improved thermostability of the fusion proteins might make them more tolerant of heat treatments used in production of animal feed pellets. After being kept at 4 °C, all the new fusion proteins had greater activity than the published fusion protein, PlyGVE2CpCWB, which under current nomenclature is GVE2_CAT_-CP26F_CWB_. The thermophile derived lysins and their corresponding CAT domains had little to no activity against Cp39 cells (Supplementary Figure S2). 

### 2.6. Activity of the Chimeric Lysins against Pathogenic C. perfringens

Examination of the activity of these lysins against both chicken (Cp509 and Cp734) and swine (JGS1504 and JGS1659) pathogenic *C. perfringens* can be seen in Table 1. The strains Cp509 and Cp734 were isolated from chickens with necrotic enteritis, while the JGS1504 and JGS1659 strains were isolated from pigs with enteritis. The PlyGVE2 (parental thermophile lysin) and GVE2_CAT_ (CAT domain alone) proteins had little to no activity against the five tested *C. perfringens* strains. The addition of the CWB domains to this CAT domain in fusion proteins drastically improved the killing of *C. perfringens*. Interestingly, while PlyGVE2 and GVE2_CAT_ had some activity against non-*C. perfringens* bacteria, the fusion proteins with the *C. perfringens* endolysin CWB domains did not kill any of the other species tested, suggesting that addition of these CWB domains increased the activity against *C. perfringens*, and reduced non-specific activity against other bacterial species. Three of the four new fusions, all had greater killing activity against the five *C. perfringens* strains than the published fusion PlyGVE2CpCWB (GVE2_CAT_-CP26F_CWB_). The sole exception to this was GVE2_CAT_-CP10_CWB,_ which had activity similar to PlyGVE2CpCWB, which could weakly kill this strain under these conditions (lysin at 0.005 mg/mL). 

The CAT domain of PlyGspY4 is a glucosaminidase domain (by homology screening), and is predicted to cleave a different peptidoglycan bond than the CAT domains of PlyGVE2 and PlyGspY412, which have L-alanine amidase domain homology. The Y4_CAT_ fusions displayed overall reduced activity, compared to the other fusion proteins. The fusions Y4_CAT_-CP10_CWB_, Y4_CAT_-CP18_CWB_, Y4_CAT_-CP33_CWB_, and Y4_CAT_-CP41_CWB_ all had activity against the five *C. perfringens* strains tested. Y4_CAT_-CP26F_CWB_ had poor lytic activity against Cp509, and was not active against the other four *C. perfringens* strains (lysin at 0.005 mg/mL). 

A minimal inhibitory concentration (MIC) test of the more active chimeric lysins was performed by microbroth serial dilution. Interestingly, Y4_CAT_-CP41_CWB_, which was not the most active chimeric lysin by turbidity reduction assay, was the most active by the MIC assay against Cp39 bacteria (Table 2), with MIC values ranging from 1.6 to 6.3 µg/mL. Additionally, the other Y4_CAT_-containing lysin tested, Y4_CAT_-CP33_CWB_ had MIC values ranging from 25 to 100 µg/mL, whereas the Y412_CAT_-containing lysins tested, Y412_CAT_-CP33_CWB_ and Y412_CAT_-CP41_CWB_ had 100 to >100 µg/mL MIC values in independent assays. 

## 3. Discussion

We were able to identify several endolysins from the genomes of thermophilic bacteria in the IMG database. Two of these, PlyGspY412 and PlyGspY4, along with the previously identified PlyGVE2 [29,34], provided the CAT domains that we fused to *C. perfringens* CWB domains from previously reported enzymes PlyCP10, PlyCP41, PlyCP33, PlyCP18, and PlyCP26F ([30,31] and US patent application 20180195055). All fusions did not show equal activity or thermostability. Those thermophile CAT domains fused to CP41_CWB_, CP33_CWB_, and CP18_CWB_ were more active than the other chimeras in lysing *C. perfringens* (Figure 6 and Figure 7). This activity is similar to what is seen for the native *C. perfringens* lysins, with PlyCP10 having less activity than PlyCP41 [31], or than PlyCP33 and PlyCP18 (data not shown, U.S. patent application 20180195055). 

Thermostability was assessed by incubation at elevated temperatures for 15 min, then cooling, followed by assaying residual activity at 40 °C, a temperature close to the body temperature of chickens (40–43 °C), pigs (39–40 °C), and cows (38–39 °C) [35]. Steam conditioning of animal feed ingredients at 95 °C is less than 15 min, and is reported to be between 20 s and 4 min by one vendor of feed pellet milling machinery [20]. The more stringent 15 min exposure used in this study reflects the steam conditioning plus the cooling down period afterwards. After incubation at 60 °C, PlyCP18 lost activity. In previous studies of the other native *C. perfringens* lysins, PlyCP10, PlyCP41, and PlyCP26F were inactivated after exposure to temperatures between 50 °C and 60 °C [22,31]. PlyCP33 was inactivated after a 60 °C incubation (data not shown). Another native CP lysin, PlyCM was reported to have retained activity after a 30 min incubation at 50 °C, but to have lost activity after incubation at 55 °C [36]. The GVE2_CAT_- and Y412_CAT_-containing lysin chimeras showed the greatest increases in thermostability over the native endolysins. Of the Y4_CAT_-containing chimeras, only Y4_CAT_-CP33_CWB_ and Y4_CAT_-CP41_CWB_ were more stable than PlyCP18. Only the GVE2_CAT_-fusions were able to retain some low-level activity after a 15 min incubation at the more elevated temperatures of 80 °C and 95 °C, common in feed pellet processing. In fact, GVE2_CAT_-CP26F_CWB_ had fairly good stability across the board (Figure 8C), but it suffered from low activity at physiological temperatures (Figure 6 and Figure 7), whereas, GVE2_CAT_-CP41_CWB_ had good activity at physiological temperatures (Figure 6 and Figure 7), but activity decreased as treatment temperatures increased (Figure 8C), though it still retained >14% of its own activity after incubation at 95 °C (Supplementary Table S1). There are other thermostable endolysins besides those presented here, and some are very thermostable, like the Ts2631 endolysin, from *Thermus scotoductus* phage vB_Tsc2631, which retains > 80% activity after 30 min at 95 °C, and has a thermal melt-point (Tm), representing 50% unfolded, of 99.8 °C [37]. However, this endolysin was ineffective against *Clostridium sporogenes*, and had relatively low activity against other mesophilic Gram-positive bacteria when compared to its activity against *Thermus scotoductus*. Additionally, it is less active at physiological temperatures. The non-thermophile bacteriophage T5 endolysin is also very thermostable, retaining ~65% of its activity after a 30 min incubation at 90 °C [38]. Recently, a report of a new *C. perfringens* lysin, LysCPS2, indicates that LysCPS2 had 30% residual activity after incubation at 95 °C for 10 min [39]. While this incubation time was 33% shorter than our work, its stability is still impressive for a native *C. perfringens* lysin. 

It is known by the work of Kusuma et al., 2005 [33], that peptidoglycan hydrolase assays do not always agree quantitatively. We too find that relative activity of our fusions is not always maintained between turbidity reduction vs. plate lysis vs. MIC assays. Although this can be confounding when analyzing the data, it has the advantage of identifying candidate enzymes for further study that might otherwise have been overlooked. Both plate lysis and TRA did not suggest that Y4_CAT_-CP41_CWB_ was going to stand out, with almost 10-fold increased relative activity in the MIC assay. In fact, both Y4_CAT_-CP41_CWB_ and Y4_CAT_-CP33_CWB_ chimeras showed substantially more activity than the other lysins. Because of this MIC data, the Y4_CAT_-CP41_CWB_ lysin and, to a lesser extent, the Y4_CAT_-CP33_CWB_ lysin could still have potential as a therapeutic drug for the treatment of animals or humans. The absence of activity in the MIC assay for the GVE2_CAT_-containing lysins, GVE2_CAT_-CP33_CWB_ and GVE2_CAT_-CP41_CWB_, was very interesting when compared to how active they are in the TRA and the plate lysis assay. It could be that they are inhibited under the conditions of the MIC assay. Unlike PlyGVE2 and PlyGspY412, which have an L-alanine amidase CAT domain, the CAT domain of PlyGspY4 is predicted to be a glucosaminidase. Possibly, this targeting of a different bond in the peptidoglycan is why chimeras containing this domain were more effective in the MIC assay. No glucosaminidase-containing endolysins were found in a 2011 study which analyzed nine public *C. perfringens* genomes, and found 45 endolysin-like enzymes [36]. This suggests the Y4_CAT_ fusions may represent a rare lytic activity against *C. perfringens* strains. 

The goal of this study was to identify optimal fusions for further testing in animal/human model systems. Thus, relative activity levels are sufficient for the goals of this work. Our rationale for performing the peptidoglycan hydrolase assays with µg/mL enzyme concentrations, rather than molar concentrations, was two-fold. First, so we would generate results that are more readily comparable between assays and, second, to follow convention (the MIC assay results are always expressed in µg/mL). This raises the caveat that, if the size of the enzymes are highly variable, this could yield results that might be misleading (e.g., if comparing enzymes with 10× different MW: at equal µg/mL concentrations, there are 10× more enzyme molecules of the smaller construct than the 10× larger MW constructs). The validity of our approach to identify our top candidates is supported by the fact that our constructs are all similarly sized [none of the constructs have more than 2–3-fold difference in MW (see MWs in Figure 3, Figure 4 and Figure 5)], and the fact that the most active constructs in each series were the larger MW constructs (which represent the smaller number of enzyme molecules).

Unexpectedly, the native thermophile lysins and their CAT domains without CWBs were active against *Bacillus cereus* (Table 1). The fusion of *C. perfringens* CWBs to the CAT domains eliminated this activity. This phenomenon suggests that these native thermophile endolysins might be targeted against *B. cereus* or related species without a CWB domain swap. Random or directed point mutagenesis may prove sufficient to increase the effectiveness of these lysins against *B. cereus.*

The ability of these thermophile-derived CAT domains to lyse *C. perfringens* strains when fused to a variety CWB domains from *C. perfringens* endolysins suggests that they would also be active when fused to other binding domains that target different species of Gram-positive bacteria, though several binding domains may need to be screened to find effective CAT–CWB fusions. 

## 4. Materials and Methods

### 4.1. Endolysin Synthetic Genes, Chimeric Lysins, and Cloning Vector

The genes for the Geobacillus thermophile endolysins, PlyGsPY412 (Genbank ACX77322; predicted N-acetylmuramoyl-L-alanine amidase enzyme activity, EC 3.5.1.28), PlyGspY4 (ADP73474; predicted endo-beta-N-acetylglucosamidase enzyme activity, EC 3.2.1.96), and PlyGVE2 (YP_001285830; predicted N-acetylmuramoyl-L-alanine amidase enzyme activity, EC 3.5.1.28), were synthesized as *E. coli*-codon optimized DNA sequences and cloned into NdeI and XhoI sites of the *E. coli* expression vector pET-21a(+) by GenScript (Piscataway, NJ, USA). This resulted in the addition of a C-terminal His Tag (LEHHHHHH) to the recombinant protein sequence. The DNA sequences for the CAT domains of these enzymes and the DNA sequences for the CWB domains from the *Clostridium perfringens* endolysins PlyCP10, PlyCP18, PlyCP33, PlyCP41, and PlyCP26F were PCR amplified and ligated into the pET-21a (+) vector. The Genbank accessions numbers for the *C. perfringens* lysins are as follows: PlyCP26F (AEA86246), PlyCP10 (AQS60701), PlyCP18 (AQS60703), PlyCP33 (AQS60704), and PlyCP41 (AQS60702). All plasmids were sequenced to confirm identity and cloning integrity. Due to the presence of an *Xho*I site internal to the PlyCP18 sequence, its CWB was cloned, using a SalI site, to the plasmids *Xho*I site, which resulted in its C-terminal His Tag peptide being VEHHHHHH. The peptide sequences for the chimeric lysins are available in Supplementary Figure S1 (and in a patent application, pending at the United States Patent and Trademark Office). 

### 4.2. Bacteria Strains, Growth, and Protein Expression

*C. perfringens* strains Cp39, Cp509, Cp734, and *Bacillus cereus* strain Bc17 were from Bruce Seal, Poultry Microbiology Safety Research Unit, Agricultural Research Service, U.S. Department of Agriculture (USDA), Athens, GA. *C. perfringens* strains JGS1504 and JGS1659 were from Nancy Cornick, Veterinary Microbiology and Preventive Medicine, Iowa State University, Ames, IA. *Enterococcus faecalis* strain EF-17 was provided by Paul Hyman (Biology & Toxicology Department, Ashland University, Ashland, OH, USA). *Clostridium difficile* strain ATCC 700057 was from the American Type Culture Collection (Manassas, VA, USA). The *Streptococcus agalactiae* and *Staphylococcus aureus* strains were from Max J. Paape, USDA, Beltsville, MD.

NEB-5α *E. coli* were transformed for the purposes of cloning and plasmid DNA production. The pET-21a (+) variants were transformed into BL21(DE3) *E. coli* (Invitrogen) for the purpose of recombinant protein expression. BL21(DE3) transformants were grown in 1 liter modified Luria-Bertani (mLB) medium (15 g/L tryptone, 8 g/L yeast extract, 5 g/L NaCl) [40] with 150 µg/mL ampicillin, to exponential phase, A_600_ = 0.6, and expression was induced with 1 mM isopropyl-β-D-1-thiogalactopyranoside (IPTG) at 10 °C with shaking overnight. Cells were pelleted by centrifugation and lysed in Lysis Buffer (50 mM NaH_2_PO_4_, 300 mM NaCl, 10 mM imidazole, 30% glycerol, pH 8.0) by sonication. The lysate was then centrifuged 9000× *g* for 30 min and the supernatant was then applied to 1 mL of nickel-NTA Superflow resin (Qiagen, Valencia, CA, USA) in a column. The column was washed with 20 mL of Lysis buffer and with 40 mL Wash Buffer (50 mM NaH_2_PO_4_, 300 mM NaCl, 20 mM imidazole, 30% glycerol, pH 8.0). Recombinant protein was eluted with Elution buffer (50 mM NaH_2_PO_4_, 300 mM NaCl, 250 mM imidazole, 30% glycerol, pH 8.0). The purified proteins were stored at 4 °C (one week) and at −80 °C (long term storage). Protein purity was examined by SDS-PAGE, using 15% gels and Coomassie Blue staining.

### 4.3. Determination of Lytic Activity of the Recombinant Proteins

The turbidity reduction assay (TRA) measures the rate of reduction in the optical density (OD) or absorbance (A) at 600 nm of a suspension of *C. perfringens* bacteria, due to the lytic activity of the lysin proteins. The recombinant lysins were diluted into 50 mM NaH_2_PO_4_ pH 7.0 and mixed 1:1 with mid-log phase *C. perfringens* cells (strain Cp39) in water. The assay was conducted in a 96-well flat-bottom polystyrene plate, at 40 °C, using a Molecular Devices (Molecular Devices, LLC, San Jose, CA, USA) plate reader. Turbidity was read every 20 seconds as optical density (OD) at 600 nm over 20 min. The decrease in OD represents lysis of cells in suspension. TRA data presented is the reduction in OD after subtracting the drop in OD by cells alone, in just buffer. Each species has a small, but measurable, reduction in OD in the TRA buffer alone, presumably due to species-specific physiology, related to osmotolerance of the TRA buffer. Final enzyme concentration in the assay was 0.005 mg/mL. Activity is defined as the maximal rate of reduction, V, in the linear portion of the graph trace. Relative activity is set to the maximal rate determined for each experiment. Data represents duplicate samples in each of the three independent experiments. 

The thermostability assay measures residual activity of lysins incubated at target temperatures, prior to cooling and testing by TRA. Enzymes were incubated at 4 °C, 50 °C, 60 °C, 70 °C, 80 °C, or 95 °C for 15 min, then placed on ice for 10 min, followed by mixing the enzymes 1:1 with Cp39 cells. Residual enzyme activity was assayed by turbidity reduction in the plate reader at 40 °C, as described above.

The plate lysis assay, also known as the spot lysis assay, was performed essentially as described by Becker et al. [41]. *C. perfringens* cultures were grown to mid-log phase (OD_600_ = 0.4–0.6) in 100 mL BYC broth (37 g/L brain heart infusion, 5 g/L yeast extract, 0.5 g/L L-cysteine). Cells were pelleted by centrifugation, concentrated 50-fold in PBS 25% glycerol, and stored at −80 °C until needed. The frozen cells were thawed on ice and washed with 10 mL sterile H_2_O, and then twice more with lysin buffer A (50 mM NH_4_OAc, 10 mM CaCl_2_, 1 mM DTT, pH 6.2). The cells were then pelleted and suspended in 1 mL lysin buffer A. Twelve milliliters of melted 50 °C semisolid BYC agar (BYC with 7 g agar per liter, autoclaved) were added to the cells and then the mixture was poured into a sterile square petri dish. After solidifying, 10 µg of purified chimeric recombinant lysin, in a 5 µL volume, was spotted onto the plate. The plate was incubated in an anaerobic chamber for 2 h at 37 °C before scoring for clear zones. Active lysins create a clear zone in the turbidity of the embedded cells.

The Minimal Inhibitory Concentration (MIC) assays was done to test the effectiveness of the lysins to inhibit growth of the bacteria in rich medium. *Clostridium perfringens* strain Cp39 was grown to mid-log phase in BYCT broth (37 g/L brain heart infusion, 5 g/L yeast extract, 0.5 g/L L-cysteine, 0.5 g/L sodium thioglycolate). Thioglycolate was added to the broth to stabilize *C. perfringens* for the course of the assay. Chimeric lysins in PBS containing 25% glycerol (filter sterile) were two-fold serial diluted with BYCT broth (0.1 mL + 0.1 mL) across the row of a 96-well plate. Cp39 cells were diluted to ~8 × 10^5^ cells/mL in BYCT broth and 0.1 mL cells were added to lysins. The highest lysin concentration tested was 100 micrograms per milliliter, with 0.4 mg/mL lysin diluted four-fold after the addition of cells in the first well of each row. Serial diluted PBS containing 25% glycerol served as the buffer control. The 96-well plate was incubated in an anaerobic chamber at 37 °C for 20–24 h, before being read for absorbance at 600 nm (A_600_) in a plate reader. The MIC value is the lowest concentration of lysin that creates a visually clear well in the dilution series. Three independent assays were used to determine the MIC values for the lysins.

## 5. Conclusions

Until recent bans, the practice of feeding antibiotic growth promotants to swine and poultry was the industry norm, and replacements for those antibiotics are sorely needed. These lytic enzyme fusions represent an alternative to the use of antibiotics in the treatment of *C. perfringens* in swine, poultry, or other animals, either as a growth promotant or as a therapeutic. Fusing the thermophile endolysin CAT domains with the *C. perfringens* CWB domains produced chimeric lysins that were both active against *C. perfringens* and more thermostable than the native *C. perfringens* lysins that provided the CWB domains. While all the tested chimeric lysins had activity against *C. perfringens*, some were more active than others (see summary in Table S1). The GVE2_CAT_- and Y412_CAT_-fusions showed improved thermostability compared to PlyCP18, and some of these, like GVE2_CAT_-CP41_CWB_, could be candidates for testing in animal feed pelleting conditions. Surprisingly, Y4_CAT_-CP41_CWB_ was the best enzyme by the MIC assay, almost 10× more active than any of the other fusions tested and is thus the top candidate for use as a therapeutic agent in animal and human studies, where thermostability is less of an issue. In this study, we report the successful development of thermophile chimeric lysins, with potential uses as growth promotants in animal feed or as novel therapeutics against *C. perfringens*.

## 6. Patents

This work includes material present in a pending U.S. patent application.

## Figures and Tables

**Table 1:** Activity of lysins on *C. perfringens* strains and other bacteria.

Lysin	*Clostridium perfringens*					Other Bacteria				
	Cp39	Cp509	Cp734	Cp JGS1504	Cp JGS1659	*Bacillus cereus* Bc17	*Entero-* *coccus faecalis* EF-17	*Clostridium difficile* ATCC 700057	*Strepto-* *coccus agalactiae*	*Staphylo-* *coccus aureus* 305
GVE2_CAT_-CP10_CWB_	+/−	+	++	++	++	−	−	−	−	−
GVE2_CAT_-CP18_CWB_	++	++	++	++	++	−	−	−	−	−
GVE2_CAT_-CP33_CWB_	++	++	++	+++	++	−	−	−	−	−
GVE2_CAT_-CP41_CWB_	++	++	++	+++	++	−	−	−	−	−
GVE2_CAT_-CP26F_CWB_ (PlyGVE2CpCWB)	+/−	+	+	+	+	−	−	−	−	−
GVE2_CAT_	+/−	+/−	+/−	+/−	+/−	+/−	−	+/−	−	−
PlyGVE2	+/−	−	−	+/−	+/−	+	−	−	−	+/−
Y4_CAT_-CP10_CWB_	+/−	+	+	+	+	−	−	−	−	−
Y4_CAT_-CP18_CWB_	+	+	+/−	+	+	−	−	−	−	−
Y4_CAT_-CP33_CWB_	+	+	+/−	+	+	−	−	−	−	−
Y4_CAT_-CP41_CWB_	++	+	+/−	++	++	−	−	+/−	−	−
Y4_CAT_-CP26F_CWB_	−	+/−	−	−	−	−	−	−	−	−
Y4_CAT_	−	−	−	−	−	+/−	−	−	−	−
PlyGspY4	−	−	−	−	−	+	−	−	−	−
Y412_CAT_-CP10_CWB_	+	+	++	+	+	−	−	−	−	−
Y412_CAT_-CP18_CWB_	++	+	++	++	++	−	−	−	−	−
Y412_CAT_-CP33_CWB_	++	+	++	++	++	−	−	−	−	−
Y412_CAT_-CP41_CWB_	+	+	+/−	++	+	−	−	−	−	−
Y412_CAT_-CP26F_CWB_	+	+	+	+	+	−	−	−	−	−
Y412_CAT_	-	−	−	−	−	+/−	−	−	−	−
PlyGspY412	−	−	+/−	−	+/−	++	−	−	−	+/−

Lytic activity was determined by turbidity reduction assay using 0.005 mg/mL lysin with suspensions of the indicated bacteria in reaction buffer. Vnet is the maximal velocity of the lysis reaction minus the background (buffer alone) decrease in turbidity. Stdev is the standard deviation of the replicates. Convert to + – values as follows: “−” when (Vnet – Stdev) ≤ 5, “+/−” when (Vnet-Stdev) > 5 and ≤ 15, “+” when (Vnet-Stdev) > 15 and ≤ 45, “++” when (Vnet –Stdev) > 45, ≤ 135, “+++” when (Vnet-Stdev) > 135, where “Vnet” is the maximal velocity (V in milli OD_600_/min) of the reaction minus the velocity from the buffer control reaction, and where “Stdev” is the standard deviation from the replicate reactions.

**Table 2:** Summary of Minimal Inhibitory Concentration (MIC) of chimeric lysins against Cp39 bacteria.

Chimeric Lysin	GVE2_CAT_-CP33_CWB_	GVE2_CAT_-CP41_CWB_	Y412_CAT_-CP33_CWB_	Y412_CAT_-CP41_CWB_	Y4_CAT_-CP33_CWB_	Y4_CAT_-CP41_CWB_
MIC range ^1^ (µg/mL)	>100	>100	≥100	≥100	25–100	1.6–6.3

^1^ Data from three independent MIC assays.

## Supplementary Materials

The following are available online at https://www.mdpi.com/2079-6382/8/4/214/s1, Figure S1: Protein sequences of native lysins and recombinant fusion lysins. Figure S2: Turbidity reduction assay activity of PlyGspY412, PlyGspY4, PlyGVE2, and their catalytic domains. Table S1. Summary of Data.

## References

[1] Smith L.D., Gardner M.V. (1949). The occurrence of vegetative cells of *Clostridium perfringens* in soil. J. Bacteriol..

[2] Matches J.R., Liston J., Curran D. (1974). *Clostridium perfringens* in the environment. Appl. Microbiol..

[3] Tschirdewahn B., Notermans S., Wernars K., Untermann F. (1991). The presence of enterotoxigenic *Clostridium perfringens* strains in faeces of various animals. Int. J. Food Microbiol..

[4] Songer J.G. (1996). Clostridial enteric diseases of domestic animals. Clin. Microbiol. Rev..

[5] Scallan E., Griffin P.M., Angulo F.J., Tauxe R.V., Hoekstra R.M. (2011). Foodborne illness acquired in the United States-unspecified agents. Emerg. Infect. Dis..

[6] Songer J.G., Uzal F.A. (2005). Clostridial enteric infections in pigs. J. Vet. Diagn. Investig..

[7] Jaggi M., Wollschlager N., Abril C., Albini S., Brachelente C., Wyder M., Posthaus H. (2009). Retrospective study on necrotizing enteritis in piglets in Switzerland. Schweiz. Arch. Tierheilkd..

[8] Moon H.W., Bergeland M.E. (1965). *Clostridium perfringens* type C enterotoxemia of the newborn pig. Can. Vet. J..

[9] Collins J.E., Bergeland M.E., Bouley D., Ducommun A.L., Francis D.H., Yeske P. (1989). Diarrhea associated with *Clostridium perfringens* type A enterotoxin in neonatal pigs. J. Vet. Diagn. Investig..

[10] Cooper K.K., Songer J.G., Uzal F.A. (2013). Diagnosing clostridial enteric disease in poultry. J. Vet. Diagn. Investig..

[11] Kaldhusdal M., Løvland A. (2000). The economical impact of *Clostridium perfringens* is greater than anticipated. World Poult..

[12] FDA (2013). Guidance for Industry #213, New Animal Drugs and New Animal Drug Combination Products Administered in or on Medicated Feed or Drinking Water of Food-Producing Animals: Recommendations for Drug Sponsors for Voluntarily Aligning Product Use Conditions with GFI #209.

[13] FDA FDA Announces Implementation of GFI #213, Outlines Continuing Efforts to Address Antimicrobial Resistance. https://www.fda.gov/AnimalVeterinary/NewsEvents/CVMUpdates/ucm535154.htm.

[14] Schmelcher M., Donovan D.M., Loessner M.J. (2012). Bacteriophage endolysins as novel antimicrobials. Future Microbiol..

[15] Fischetti V.A. (2010). Bacteriophage endolysins: A novel anti-infective to control gram-positive pathogens. Int. J. Med. Microbiol..

[16] Totte J., de Wit J., Pardo L., Schuren F., van Doorn M., Pasmans S. (2017). Targeted anti-staphylococcal therapy with endolysins in atopic dermatitis and the effect on steroid use, disease severity and the microbiome: study protocol for a randomized controlled trial (MAAS trial). Trials.

[17] May K.D., Wells J.E., Maxwell C.V., Oliver W.T. (2012). Granulated lysozyme as an alternative to antibiotics improves growth performance and small intestinal morphology of 10-day-old pigs. J. Anim. Sci..

[18] Oliver W.T., Wells J.E. (2015). Lysozyme as an alternative to growth promoting antibiotics in swine production. J. Anim. Sci. Biotechnol..

[19] Cutlip S.E., Hott J.M., Buchanan N.P., Rack A.L., Latshaw J.D., Moritz J.S. (2008). The Effect of steam-conditioning practices on pellet quality and growing broiler nutritional value. J. Appl. Poult. Res..

[20] Buhler-AG Pelleting. https://www.buhlergroup.com/content/buhlergroup/global/en/products/pellet_mill.html.

[21] Mao J., Schmelcher M., Harty W.J., Foster-Frey J., Donovan D.M. (2013). Chimeric Ply187 endolysin kills *Staphylococcus aureus* more effectively than the parental enzyme. FEMS Microbiol. Lett..

[22] Swift S.M., Seal B.S., Garrish J.K., Oakley B.B., Hiett K., Yeh H.Y., Woolsey R., Schegg K.M., Line J.E., Donovan D.M. (2015). A thermophilic phage endolysin fusion to a *Clostridium perfringens*-specific cell wall binding domain creates an anti-clostridium antimicrobial with improved thermostability. Viruses.

[23] Leyh-Bouille M., Bonaly R., Ghuysen J.M., Tinelli R., Tipper D. (1970). LL-diaminopimelic acid containing peptidoglycans in walls of *Streptomyces sp*. and of *Clostridium perfringens* (type A). Biochemistry.

[24] Oliveira H., Melo L.D., Santos S.B., Nobrega F.L., Ferreira E.C., Cerca N., Azeredo J., Kluskens L.D. (2013). Molecular aspects and comparative genomics of bacteriophage endolysins. J. Virol..

[25] Scheffers D.J., Pinho M.G. (2005). Bacterial cell wall synthesis: new insights from localization studies. Microbiol. Mol. Biol. Rev..

[26] Marchler-Bauer A., Bryant S.H. (2004). CD-Search: protein domain annotations on the fly. Nucleic Acids Res..

[27] Brumm P., Land M.L., Hauser L.J., Jeffries C.D., Chang Y.-J., Mead D.A. (2015). Complete genome sequences of Geobacillus sp. Y412MC52, a xylan-degrading strain isolated from obsidian hot spring in Yellowstone National Park. Stand Genom. Sci..

[28] Liu B., Wu S., Song Q., Zhang X., Xie L. (2006). Two novel bacteriophages of thermophilic bacteria isolated from deep-sea hydrothermal fields. Curr. Microbiol..

[29] Ye T., Zhang X. (2008). Characterization of a lysin from deep-sea thermophilic bacteriophage GVE2. Appl. Microbiol. Biotechnol..

[30] Simmons M., Donovan D.M., Siragusa G.R., Seal B.S. (2010). Recombinant expression of two bacteriophage proteins that lyse Clostridium perfringens and share identical sequences in the C-terminal cell wall binding domain of the molecules but are dissimilar in their N-terminal active domains. J. Agric. Food Chem..

[31] Swift S.M., Waters J.J., Rowley D.T., Oakley B.B., Donovan D.M. (2018). Characterization of two glycosyl hydrolases, putative prophage endolysins, that target *Clostridium perfringens*. FEMS Microbiol. Lett..

[32] Potter S.C., Luciani A., Eddy S.R., Park Y., Lopez R., Finn R.D. (2018). HMMER web server: 2018 update. Nucleic Acids Res..

[33] Kusuma C., Kokai-Kun J. (2005). Comparison of four methods for determining lysostaphin susceptibility of various strains of *Staphylococcus aureus*. Antimicrob. Agents Chemother..

[34] Jin M., Ye T., Zhang X. (2013). Roles of bacteriophage GVE2 endolysin in host lysis at high temperatures. Microbiology.

[35] Robertshaw D., Reece W.O. (2004). Temperature regulation and thermal environment. Dukes’ Physiology of Domestic Animals.

[36] Schmitz J.E., Ossiprandi M.C., Rumah K.R., Fischetti V.A. (2011). Lytic enzyme discovery through multigenomic sequence analysis in *Clostridium perfringens*. Appl. Microbiol. Biotechnol..

[37] Plotka M., Kaczorowska A.K., Morzywolek A., Makowska J., Kozlowski L.P., Thorisdottir A., Skirnisdottir S., Hjorleifsdottir S., Fridjonsson O.H., Hreggvidsson G.O. (2015). Biochemical characterization and validation of a catalytic site of a highly thermostable Ts2631 endolysin from the thermus scotoductus phage vB_Tsc2631. PLoS ONE.

[38] Shavrina M.S., Zimin A.A., Molochkov N.V., Chernyshov S.V., Machulin A.V., Mikoulinskaia G.V. (2016). In vitro study of the antibacterial effect of the bacteriophage T5 thermostable endolysin on *Escherichia coli* cells. J. Appl. Microbiol..

[39] Ha E., Son B., Ryu S. (2018). Clostridium perfringens virulent bacteriophage CPS2 and its thermostable endolysin LysCPS2. Viruses.

[40] Schmelcher M., Powell A.M., Becker S.C., Camp M.J., Donovan D.M. (2012). Chimeric phage lysins act synergistically with lysostaphin to kill mastitis-causing Staphylococcus aureus in murine mammary glands. Appl. Environ. Microbiol..

[41] Becker S.C., Dong S., Baker J.R., Foster-Frey J., Pritchard D.G., Donovan D.M. (2009). LysK CHAP endopeptidase domain is required for lysis of live staphylococcal cells. FEMS Microbiol. Lett..

