# Supplementary material for: Thermophile Lytic Enzyme Fusion Proteins that Target Clostridium perfringens
Source: Antibiotics (Basel). 2019 Nov 8;8(4):214. doi: 10.3390/antibiotics8040214 (PMC6963370; doi:10.3390/antibiotics8040214)
Supplement: Supplementary file 1 [file antibiotics-08-00214-s001.zip › Table S1.docx]

**Table S1.** Summary of Data.

| **Source** | **Figure 7 ^a^** | **Table 1 ^b^** | **Figure 8 ^c^. Thermostability** | | | | **Derived from Figure 8 ^c^** | | **Table 2** |
| --- | --- | --- | --- | --- | --- | --- | --- | --- | --- |
| **Lysin** | **Plate Lysis Assay vs. Cp39**  **(0-5) *^a^*** | **Average activity vs. 5 *C.p.* strains**  **(0-3) *^b^*** | **Residual activity after 4 °C incubation*^C^*** | **Residual activity after 60 °C incubation*^C^*** | | **Residual activity after 95 °C incubation *^C^*** | **Residual activity after 60 °C, relative to self after 4°C (100%)** | **Residual activity after 95 °C, relative to self after 4°C (100%)** | **MIC (µg/mL)** |
| **GVE2_CAT_-CP10_CWB_** | 3 | 1.5 | 21.3 | 18.6 | 2.1 | | 87.3 | 9.9 | n.d. |
| **GVE2_CAT_-CP18_CWB_** | 4 | 2 | 58.2 | 36.6 | 3.6 | | 62.9 | 6.2 | n.d. |
| **GVE2_CAT_-CP33_CWB_** | 5 | 2.2 | 68.5 | 22.3 | 1.7 | | 32.6 | 2.5 | >100 |
| **GVE2_CAT_-CP41_CWB_** | 5 | 2.2 | 84.1 | 73.2 | 12.1 | | 87.0 | 14.4 | >100 |
| **GVE2_CAT_-CP26F_Cwb_** | 2 | 0.9 | 13 | 13.5 | 4.8* | | 103.8 | 36.9* | n.d. |
| **GVE2_CAT_** | 0 | 0.5 | n.d. | n.d. | n.d. | | n.d. | n.d. | n.d. |
| **PlyGVE2** | n.d. | 0.3 | n.d. | n.d. | n.d. | | n.d. | n.d. | n.d. |
|  |  |  |  |  |  | |  |  |  |
| **Y4_CAT_-CP10_CWB_** | 2 | 0.9 | 4.4 | 1.6 | 0 | | 36.4 | 0.0 | n.d. |
| **Y4_CAT_-CP18_CWB_** | 3 | 0.9 | 8.7 | 2.3 | 1.1 | | 26.4 | 12.6 | n.d. |
| **Y4_CAT_-CP33_CWB_** | 4 | 0.9 | 26.6 | 7.1 | 0.2 | | 26.7 | 0.8 | 25 - 100 |
| **Y4_CAT_-CP41_CWB_** | 4 | 1.5 | 44.5 | 26.9 | 0.5 | | 60.4 | 1.1 | 1.6 - 6.3 |
| **Y4_CAT_-CP26F_CWB_** | 1 | 0.1 | 0 | 0 | 0 | | 0.0 | 0.0 | n.d. |
| **Y4_CAT_** | 0 | 0 | n.d. | n.d. | n.d. | | n.d. | n.d. | n.d. |
| **PlyGspY4** | n.d. | 0 | n.d. | n.d. | n.d. | | n.d. | n.d. | n.d. |
|  |  |  |  |  |  | |  |  |  |
| **Y412_CAT_-CP10_CWB_** | 2 | 1.2 | 32 | 27.2 | 0 | | 85.0 | 0.0 | n.d. |
| **Y412_CAT_-CP18_CWB_** | 4 | 1.8 | 65.9 | 42.5 | 0 | | 64.5 | 0.0 | n.d. |
| **Y412_CAT_-CP33_CWB_** | 5 | 1.8 | 90.8 | 19.4 | 0 | | 21.4 | 0.0 | 100 - >100 |
| **Y412_CAT_-CP41_CWB_** | 5 | 1.1 | 31 | 15.8 | 0.4 | | 51.0 | 1.3 | 100 - >100 |
| **Y412_CAT_-CP26F_CWB_** | 2 | 1 | 25.6 | 26.7 | 0.1 | | 104.3 | 0.4 | n.d. |
| **Y412_CAT_** | 0 | 0 | n.d. | n.d. | n.d. | | n.d. | n.d. | n.d. |
| **PlyGspY412** | n.d. | 0.2 | n.d. | n.d. | n.d. | | n.d. | n.d. | n.d. |
|  |  |  |  |  |  | |  |  |  |
| **PlyCP18** | n.d. | n.d. | **100** | **0** | **0** | | **0.0** | **0.0** | n.d. |

*a* Figure 7, ranking of clear zones from lysins spotted onto Cp39 cells, with 5 being largest clearing (most active) to zero being no clearing (least active). *b* Table 1, average activity by TRA of 5 *C. perfringens* strains, with 3 being most active and zero being inactive. *c* Figure 8, Residual activity after a 15 min incubation at the target temperature relative to the PlyCP18 activity from 4 °C treatment, with TRA done at 40 °C. * Relatively large error bars at 95°C, see Figure 8C.
