# Supplementary figures and images for: Thermophile Lytic Enzyme Fusion Proteins that Target Clostridium perfringens
Source: Antibiotics (Basel). 2019 Nov 8;8(4):214. doi: 10.3390/antibiotics8040214 (PMC6963370; doi:10.3390/antibiotics8040214)

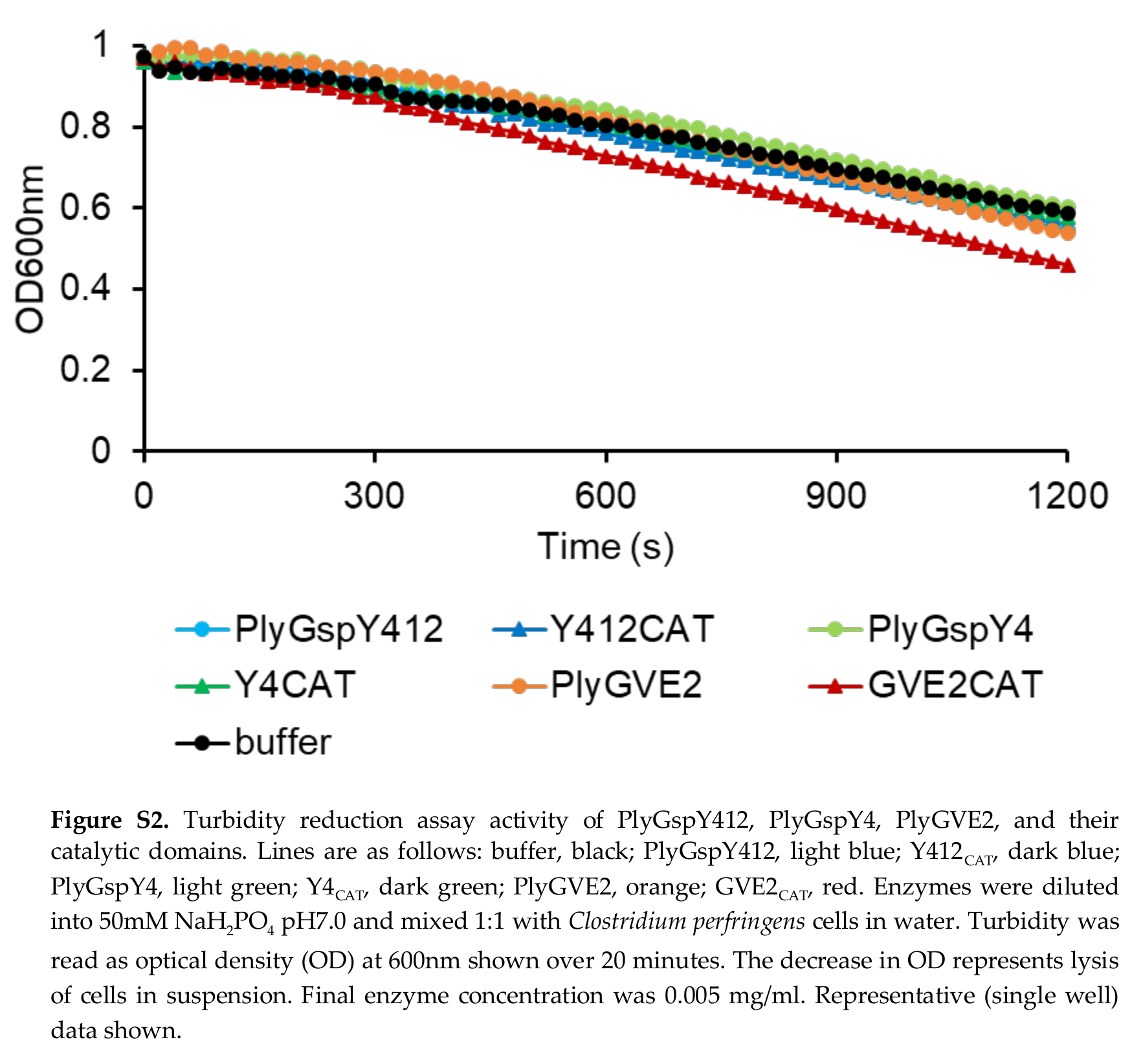

Supplement: Supplementary file 1 [file antibiotics-08-00214-s001.zip › supplementary Figure S2.tif]
