# Supplementary material for: Thermophile Lytic Enzyme Fusion Proteins that Target Clostridium perfringens
Source: Antibiotics (Basel). 2019 Nov 8;8(4):214. doi: 10.3390/antibiotics8040214 (PMC6963370; doi:10.3390/antibiotics8040214)
Supplement: Supplementary file 1 [file antibiotics-08-00214-s001.zip › supplementary Figure S1.doc]

>PlyGspY412 (native protein at NCBI: ACX77322.1 cell wall hydrolase/autolysin [Geobacillus sp. Y412MC61])

MMVRIVLDAGHGGHDPGAVANGLREKDLTLAIVKHIGKMLGEYEGAEVHY

TRTDDRFLELSERAAIANKLKADLLISVHINAGGGTGFESYIYNGNVSPA

TIAYQNVIHQELMKAIGNVTDRGKKRANYAVLRETNMPAILTENLFIDNA

NDAAKLKSEQFLQQVAYGHVQGIVKAFGLKKKAKPQTKQKVSDGKLYRVQ

VGAFADPENAKRLADELKKKGYPATIVLEHHHHHH

>Y412_cat_

MMVRIVLDAGHGGHDPGAVANGLREKDLTLAIVKHIGKMLGEYEGAEVHYTRTDDRFLEL

SERAAIANKLKADLLISVHINAGGGTGFESYIYNGNVSPATIAYQNVIHQELMKAIGNVT

DRGKKRANYAVLRETNMPAILTENLFIDNANDAAKLKSEQFLQQVAYGHVQGIVKAFGSS

LEHHHHHH

>Y412_cat_-CP10_cwb_

MMVRIVLDAGHGGHDPGAVANGLREKDLTLAIVKHIGKMLGEYEGAEVHYTRTDDRFLEL

SERAAIANKLKADLLISVHINAGGGTGFESYIYNGNVSPATIAYQNVIHQELMKAIGNVT

DRGKKRANYAVLRETNMPAILTENLFIDNANDAAKLKSEQFLQQVAYGHVQGIVKAFGSS

LDGSTQEIFINGASQKATENKSFFTNARAKVALDPRSNPSDNYKDLGEIYAEERIQVLAE

ICDREDYLPVKYWKDASGCESSKVWVNANKDYLEIDTNARSFNIVTELDARYEPSVNSKR

MGYVKNNERLYVHRVEGDYVLATYYAGNGYKTAWFTKEYIIKDLEHHHHHH

>Y412_cat_-CP18_cwb_

MMVRIVLDAGHGGHDPGAVANGLREKDLTLAIVKHIGKMLGEYEGAEVHYTRTDDRFLEL

SERAAIANKLKADLLISVHINAGGGTGFESYIYNGNVSPATIAYQNVIHQELMKAIGNVT

DRGKKRANYAVLRETNMPAILTENLFIDNANDAAKLKSEQFLQQVAYGHVQGIVKAFGSS

LERMLKSIDENIVNDTDTTDVPSSDDSNKKDFSTNARALVALDPRDNPSDNYSDLGEIYK

DERFRVLAEVCDKGDFLPIVYWKDSEGRESGKVWVRSKQDYMMIDTYHKVFNVITELDAR

YEPSPNSSRMGYVTNGERLYVHRIEGNYALATYFAGNGYKTAWFTKKYIEKIVEHHHHHH

>Y412_cat_-CP33_cwb_

MMVRIVLDAGHGGHDPGAVANGLREKDLTLAIVKHIGKMLGEYEGAEVHYTRTDDRFLEL

SERAAIANKLKADLLISVHINAGGGTGFESYIYNGNVSPATIAYQNVIHQELMKAIGNVT

DRGKKRANYAVLRETNMPAILTENLFIDNANDAAKLKSEQFLQQVAYGHVQGIVKAFGSS

LDGSLSEFKNNSYRPTGGSSETVVSENGFYTSNEERTNATIVGKGDIEVLDEKGKVIQGR

HISSLDRVFVLGIYPSRNHIELIYPGKDEKYHAYISIENYSRLSFDYHMQYKNDDGVTYV

WWDSKNVNVKNHDEELQPHQKASPMYRTNGWLRVTFYRADGNPSDGYVRYEGEQKERFYR

KGKVVNVRTSLTVRAGAGTNYSAIGSLDPNENVEILEKTEGWYYIEYNARNERKRGYVSK

KYIEIIQLEHHHHHH

>Y412_cat_-CP41_cwb_

MMVRIVLDAGHGGHDPGAVANGLREKDLTLAIVKHIGKMLGEYEGAEVHYTRTDDRFLEL

SERAAIANKLKADLLISVHINAGGGTGFESYIYNGNVSPATIAYQNVIHQELMKAIGNVT

DRGKKRANYAVLRETNMPAILTENLFIDNANDAAKLKSEQFLQQVAYGHVQGIVKAFGSS

LDGSEDFLKKDFTLENATTCNVDTELNIRAKGTTGATIVGSIPAGDRFRIKWVDSDYLGW

YYIEYQGITGYVSQDYVEKLQMATTCNVDSVLNVRAEGNTSSNIVATINPGEVFRIDWVD

SDFIGWYRITTANGANGFVKSDFVKKLLEHHHHHH

>Y412_cat_-CP26F_cwb_

MMVRIVLDAGHGGHDPGAVANGLREKDLTLAIVKHIGKMLGEYEGAEVHYTRTDDRFLEL

SERAAIANKLKADLLISVHINAGGGTGFESYIYNGNVSPATIAYQNVIHQELMKAIGNVT

DRGKKRANYAVLRETNMPAILTENLFIDNANDAAKLKSEQFLQQVAYGHVQGIVKAFGSS

LDGSRYLANAIDPNIPLEKEQDYYRVCVQRFTNKEDAEKAQQRISNELGYYCFAEKILEH

HHHHH

>PlyGspY4 (native protein at NCBI: ADP73474.1 Mannosyl-glycoprotein endo-beta-N-acetylglucosaminidase [Geobacillus sp. Y4.1MC1])

MNDFIREIAPFAQRIQEKYRILASLVIAQACLESNFGQSGLAQKGKNLFG

VKGSYNGQSVTMKTTEYRGGKAYQTDAAFRKYPSWFESLDDLAKLYVNGV

SWDRNKYKPIIGETNYVIACKKVQECGYATDPNYASKLISIIEKYDLTKY

DKVGNKKPVKSAVAAKKEKPQIYIVQKGDTLTAIAKRYNTSVQNLVKLNN

IKNPDLILVGQKLRVKLEHHHHHH

>Y4_cat_

MNDFIREIAPFAQRIQEKYRILASLVIAQACLESNFGQSGLAQKGKNLFGVKGSYNGQSV

TMKTTEYRGGKAYQTDAAFRKYPSWFESLDDLAKLYVNGVSWDRNKYKPIIGETNYVIAC

KKVQECGYATDPNYASKLISIIEKYDLTKYDKVGSSLEHHHHHH

>Y4cat-CP10_cwb_

MNDFIREIAPFAQRIQEKYRILASLVIAQACLESNFGQSGLAQKGKNLFGVKGSYNGQSV

TMKTTEYRGGKAYQTDAAFRKYPSWFESLDDLAKLYVNGVSWDRNKYKPIIGETNYVIAC

KKVQECGYATDPNYASKLISIIEKYDLTKYDKVGSSLDGSTQEIFINGASQKATENKSFF

TNARAKVALDPRSNPSDNYKDLGEIYAEERIQVLAEICDREDYLPVKYWKDASGCESSKV

WVNANKDYLEIDTNARSFNIVTELDARYEPSVNSKRMGYVKNNERLYVHRVEGDYVLATY

YAGNGYKTAWFTKEYIIKDLEHHHHHH

>Y4_cat_-CP18_cwb_

MNDFIREIAPFAQRIQEKYRILASLVIAQACLESNFGQSGLAQKGKNLFGVKGSYNGQSV

TMKTTEYRGGKAYQTDAAFRKYPSWFESLDDLAKLYVNGVSWDRNKYKPIIGETNYVIAC

KKVQECGYATDPNYASKLISIIEKYDLTKYDKVGSSLERMLKSIDENIVNDTDTTDVPSS

DDSNKKDFSTNARALVALDPRDNPSDNYSDLGEIYKDERFRVLAEVCDKGDFLPIVYWKD

SEGRESGKVWVRSKQDYMMIDTYHKVFNVITELDARYEPSPNSSRMGYVTNGERLYVHRI

EGNYALATYFAGNGYKTAWFTKKYIEKIVEHHHHHH

>Y4_cat_-CP33_cwb_

MNDFIREIAPFAQRIQEKYRILASLVIAQACLESNFGQSGLAQKGKNLFGVKGSYNGQSV

TMKTTEYRGGKAYQTDAAFRKYPSWFESLDDLAKLYVNGVSWDRNKYKPIIGETNYVIAC

KKVQECGYATDPNYASKLISIIEKYDLTKYDKVGSSLDGSLSEFKNNSYRPTGGSSETVV

SENGFYTSNEERTNATIVGKGDIEVLDEKGKVIQGRHISSLDRVFVLGIYPSRNHIELIY

PGKDEKYHAYISIENYSRLSFDYHMQYKNDDGVTYVWWDSKNVNVKNHDEELQPHQKASP

MYRTNGWLRVTFYRADGNPSDGYVRYEGEQKERFYRKGKVVNVRTSLTVRAGAGTNYSAI

GSLDPNENVEILEKTEGWYYIEYNARNERKRGYVSKKYIEIIQLEHHHHHH

>Y4_cat_-CP41_cwb_

MNDFIREIAPFAQRIQEKYRILASLVIAQACLESNFGQSGLAQKGKNLFGVKGSYNGQSV

TMKTTEYRGGKAYQTDAAFRKYPSWFESLDDLAKLYVNGVSWDRNKYKPIIGETNYVIAC

KKVQECGYATDPNYASKLISIIEKYDLTKYDKVGSSLDGSEDFLKKDFTLENATTCNVDT

ELNIRAKGTTGATIVGSIPAGDRFRIKWVDSDYLGWYYIEYQGITGYVSQDYVEKLQMAT

TCNVDSVLNVRAEGNTSSNIVATINPGEVFRIDWVDSDFIGWYRITTANGANGFVKSDFV

KKLLEHHHHHH

>Y4_cat_-CP26F_cwb_

MNDFIREIAPFAQRIQEKYRILASLVIAQACLESNFGQSGLAQKGKNLFGVKGSYNGQSV

TMKTTEYRGGKAYQTDAAFRKYPSWFESLDDLAKLYVNGVSWDRNKYKPIIGETNYVIAC

KKVQECGYATDPNYASKLISIIEKYDLTKYDKVGSSLDGSRYLANAIDPNIPLEKEQDYY

RVCVQRFTNKEDAEKAQQRISNELGYYCFAEKILEHHHHHH

>PlyGVE2 (native protein at NCBI: YP_001285830.1 putative N-acetylmuramoyl-L-alanine amidase [Geobacillus virus E2])

MKKIFWDKGHGGSDPGAVANGLQEKNLTHKIVEYATDYLAAHYEGFTQRVSREGDQSLTL

DQRADMANKWGADVFVSVHINAGKGTGFEIYVHPNASPQSIALQNVLHGEILSAMRQFGN

ITDRGKKRANYAVLRETKMPAVLTENLFIDSNDAKHLKNEAFLKAVGEAHARGVAKFLGL

KEKQKAQPEAKPQQKPSDKKLYRVQVGAFADRENAERLAEELKRKGYPVYITDLEHHHHH

H

>GVE2_cat_

MKKIFWDKGHGGSDPGAVANGLQEKNLTHKIVEYATDYLAAHYEGFTQRVSREGDQSLTL

DQRADMANKWGADVFVSVHINAGKGTGFEIYVHPNASPQSIALQNVLHGEILSAMRQFGN

ITDRGKKRANYAVLRETKMPAVLTENLFIDSNDAKHLKNEAFLKAVGEAHARGVAKFLGS

SLEHHHHHH

>GVE2_cat_-CP10_cwb_

MKKIFWDKGHGGSDPGAVANGLQEKNLTHKIVEYATDYLAAHYEGFTQRVSREGDQSLTL

DQRADMANKWGADVFVSVHINAGKGTGFEIYVHPNASPQSIALQNVLHGEILSAMRQFGN

ITDRGKKRANYAVLRETKMPAVLTENLFIDSNDAKHLKNEAFLKAVGEAHARGVAKFLGS

SLDGSTQEIFINGASQKATENKSFFTNARAKVALDPRSNPSDNYKDLGEIYAEERIQVLA

EICDREDYLPVKYWKDASGCESSKVWVNANKDYLEIDTNARSFNIVTELDARYEPSVNSK

RMGYVKNNERLYVHRVEGDYVLATYYAGNGYKTAWFTKEYIIKDLEHHHHHH

>GVE2_cat_-CP18_cwb_

MKKIFWDKGHGGSDPGAVANGLQEKNLTHKIVEYATDYLAAHYEGFTQRVSREGDQSLTL

DQRADMANKWGADVFVSVHINAGKGTGFEIYVHPNASPQSIALQNVLHGEILSAMRQFGN

ITDRGKKRANYAVLRETKMPAVLTENLFIDSNDAKHLKNEAFLKAVGEAHARGVAKFLGS

SLERMLKSIDENIVNDTDTTDVPSSDDSNKKDFSTNARALVALDPRDNPSDNYSDLGEIY

KDERFRVLAEVCDKGDFLPIVYWKDSEGRESGKVWVRSKQDYMMIDTYHKVFNVITELDA

RYEPSPNSSRMGYVTNGERLYVHRIEGNYALATYFAGNGYKTAWFTKKYIEKIVEHHHHH

H

>GVE2_cat_-CP33_cwb_

MKKIFWDKGHGGSDPGAVANGLQEKNLTHKIVEYATDYLAAHYEGFTQRVSREGDQSLTL

DQRADMANKWGADVFVSVHINAGKGTGFEIYVHPNASPQSIALQNVLHGEILSAMRQFGN

ITDRGKKRANYAVLRETKMPAVLTENLFIDSNDAKHLKNEAFLKAVGEAHARGVAKFLGS

SLDGSLSEFKNNSYRPTGGSSETVVSENGFYTSNEERTNATIVGKGDIEVLDEKGKVIQG

RHISSLDRVFVLGIYPSRNHIELIYPGKDEKYHAYISIENYSRLSFDYHMQYKNDDGVTY

VWWDSKNVNVKNHDEELQPHQKASPMYRTNGWLRVTFYRADGNPSDGYVRYEGEQKERFY

RKGKVVNVRTSLTVRAGAGTNYSAIGSLDPNENVEILEKTEGWYYIEYNARNERKRGYVS

KKYIEIIQLEHHHHHH

>GVE2_cat_-CP41_cwb_

MKKIFWDKGHGGSDPGAVANGLQEKNLTHKIVEYATDYLAAHYEGFTQRVSREGDQSLTL

DQRADMANKWGADVFVSVHINAGKGTGFEIYVHPNASPQSIALQNVLHGEILSAMRQFGN

ITDRGKKRANYAVLRETKMPAVLTENLFIDSNDAKHLKNEAFLKAVGEAHARGVAKFLGS

SLDGSEDFLKKDFTLENATTCNVDTELNIRAKGTTGATIVGSIPAGDRFRIKWVDSDYLG

WYYIEYQGITGYVSQDYVEKLQMATTCNVDSVLNVRAEGNTSSNIVATINPGEVFRIDWV

DSDFIGWYRITTANGANGFVKSDFVKKLLEHHHHHH

>PlyGVE2CpCWB (GVE2_cat_-CP26F_cwb_)

MGMKKIFWDKGHGGSDPGAVANGLQEKNLTHKIVEYATDYLAAHYEGFTQRVSREGDQSL

TLDQRADMANKWGADVFVSVHINAGKGTGFEIYVHPNASPQSIALQNVLHGEILSAMRQF

GNITDRGKKRANYAVLRETKMPAVLTENLFIDSNDAKHLKNEAFLKAVGEAHARGVAKFL

GRYLANAIDPNIPLEKEQDYYRVCVQRFTNKEDAEKAQQRISNELGYYCFAEKILEHHHH

HH

**Figure S1.** Protein sequences of native lysins and recombinant fusion lysins. Recombinant lysins consist of thermophile-derive N-terminal catalytic (CAT) domains and *Clostridium perfringens* derived C-terminal cell wall binding (CWB) domains. Sequences in red represent the fusion joint linker amino acids and the C-terminal HisTag.
